# Diurnal shifts in eDNA concentration of threatened juvenile river herring

**DOI:** 10.17912/micropub.biology.001844

**Published:** 2025-11-03

**Authors:** Camden G. Bailey, Chase G. Spicer, William Langley, Cory S. Joyner, Patrick Harris, Roger A. Rulifson, Erin K. Field

**Affiliations:** 1 Biology, East Carolina University, Greenville, North Carolina, United States

## Abstract

River herring, *Alosa* species, are threatened in North Carolina waters. Environmental DNA (eDNA) is a non-invasive approach to monitor their movement. River herring, particularly juveniles, are known to exhibit diurnal shifts within water columns. To assess how this influenced the detection of eDNA concentrations from fish, water samples were collected from the Roanoke River during the day and night of the same day for four weeks. Using qPCR analyses, we found that nighttime eDNA concentrations consistently exceeded those of daytime concentrations indicating night is a better time to sample. If night sampling is infeasible, consistency in sampling time is important.

**Figure 1. Comparison of juvenile river herring eDNA concentrations between day and nighttime sampling:**
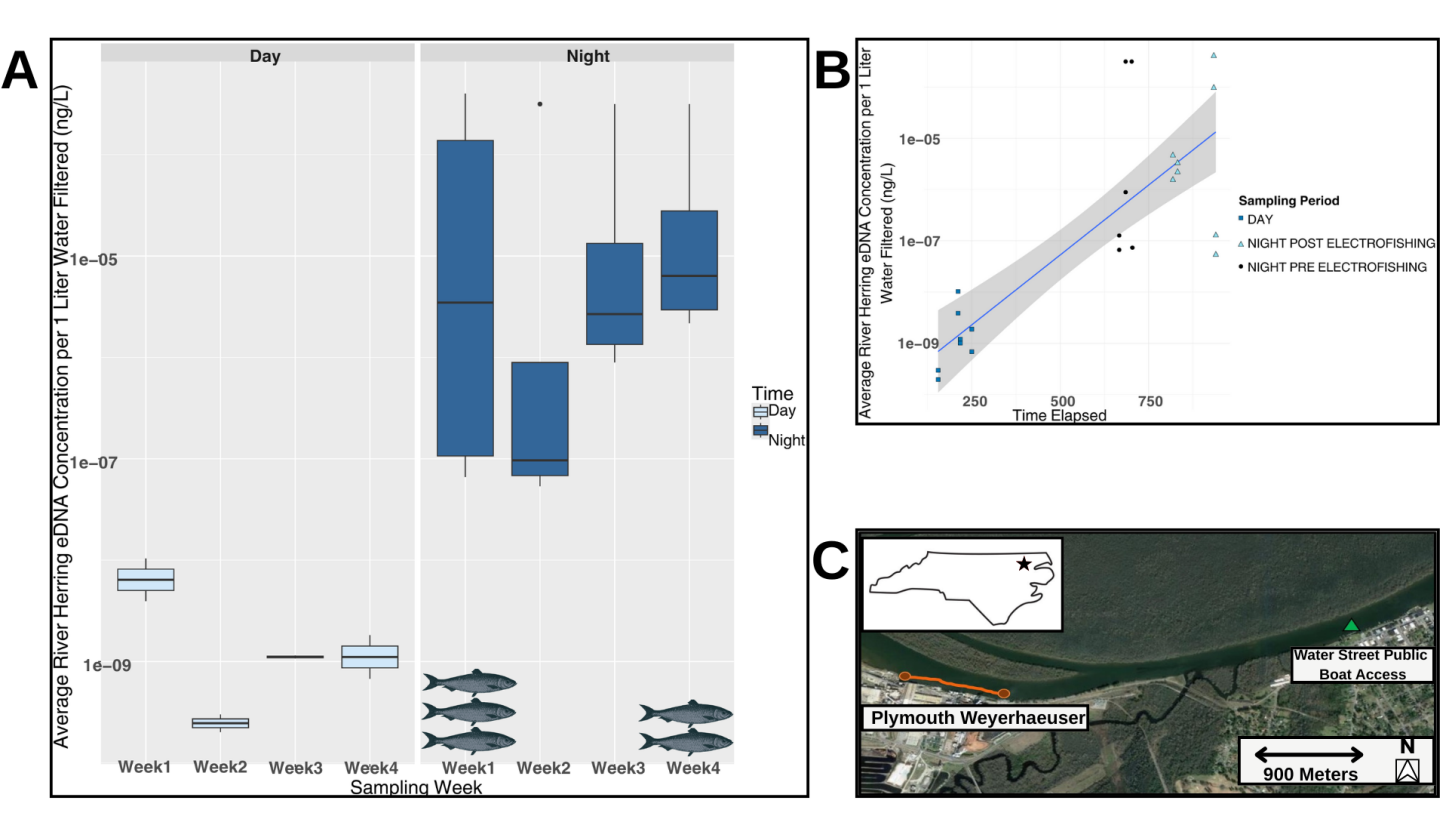
Figure 1. (A) *Alosa* spp. eDNA concentrations determined for daytime and nighttime samples over a four-week sampling period. Fish at the bottom of the plot represent river herring caught while electrofishing at night during the respective week (three fish during Week 1, two during week 4, and none during weeks 2 and 3). (B) Results indicate a significant correlation between the time sampled (time in minutes elapsed past 8:00 am) and eDNA concentrations per a fit Generalized Linear Model with the gray area representing a 95% confidence interval with a R2=0.66. (C) Sampling occurred in Plymouth, North Carolina (inset image, star) along the Roanoke River. Water samples for eDNA analyses were collected at Plymouth Water Street Public Boat Access (green triangle) and electrofishing by team members in all nighttime sampling efforts occurred near the Plymouth Weyerhaeuser (orange line).

## Description

Blueback Herring ( *Alosa aestivalis)* and Alewife ( *A. pseudoharengus)* are collectively referred to as river herring. These are anadromous fishes that spawn in freshwater environments but live in the ocean. River herring enter the Roanoke River basin through the Albemarle Sound in the spring to spawn in freshwater habitats. They are listed as threatened in North Carolina waters due to urban development, overfishing, and water quality issues (Hare et al., 2021). Many organisms, including fishes, exhibit nocturnal behaviors due to feeding strategies or anthropogenic light attractions (Perkin and Wilson 2021). Depending on the life stage of a river herring, different movements within the water column can be exhibited (Huse, 2016, Saenz-Agudelo et. al 2022).

Environmental DNA (eDNA) is DNA found in the environment from organisms shedding trace amounts of DNA in water. This DNA can be utilized to study populations without causing harm to the organisms in their natural habitat (Fowler et al., 2024). Larger fish have been shown to shed eDNA at rates three to four times higher than juvenile fish, making the detection of juveniles significantly more challenging in eDNA surveys (Maruyama et al., 2014). Recent works suggests that not taking diel vertical movements of juvenile fish into account by sampling at different times over a twenty- four-hour period could underestimate population sizes (Maes et al., 1999). As most eDNA water collections are done near the surface, the amount of eDNA recovered from juvenile river herring could be influenced by this movement. Therefore, due to variations in the movement patterns between daytime and nighttime, the goal of this study was to determine how the time of day affected the detection of eDNA concentrations from these fish (Rillahan and He, 2023). The aim of this study was to determine if the relative abundance of juvenile river herring eDNA differs between day and night samples. If significant differences are found between day and night eDNA concentrations, the annual recruitment of these species can be assayed with a better idea of when and where they are at a specific point in time to maximize results of sampling efforts for river herring management (Spear et al., 2021).

Concentrations of daytime and nighttime eDNA obtained from qPCR from a 4-week sampling period revealed that concentrations of juvenile river herring eDNA were 37,522x higher at nighttime compared to daytime concentrations ( Figure 1A ). Over all four weeks eDNA concentrations were consistently higher in nighttime samples than daytime samples, regardless of if nighttime samples were collected before or after electrofishing indicating this was a consistent trend over time ( Figure 1B ). However, there was variability in the concentration of river herring eDNA between weeks, which was likely due to movement of the juveniles out of the river system. Overall, average concentrations of *Alosa* spp. eDNA in the night samples were found to be 9.17 x 10-5 ng/1L filtered, and day samples were found to have an average concentration of 2.44 x 10-9 ng/1L filtered. Daytime and nighttime river herring eDNA concentrations were significantly different (Kruskal-Wallis Test, p<0.05), with eDNA concentrations increasing up to 1.2% per minute post-daytime sampling, per a fit Generalized Linear Model using elapsed time as the sole predictor (R2=0.66; p < 0.05) ( Figure 1B ). On September 21st, 2022, three blueback herring were documented via electrofishing, and on November 10th, 2022, two alewife juveniles were similarly recorded. This supports that the presence of eDNA was due to juvenile river herring present in the system at time of sampling and not residual eDNA from earlier in the day.

Based on these results, average concentrations of *Alosa* spp. eDNA in the night samples (9.17 x 10-5 ng/1L filtered) consistently exceeded that found in the day samples (2.44 x 10-9 ng/1L filtered) when measured on the same day. This study suggests that sampling at night, when possible, is likely to increase the likelihood of juvenile river herring detection due to higher eDNA concentrations. Additionally, no significant difference was found between the time at night at which samples were collected suggesting that the water samples for eDNA can be collected before or after electrofishing when conducted together. Because it may be impractical to sample river herring at night, it is important to at least be consistent in collecting eDNA samples at similar times of day to avoid any skewed or biased eDNA results when studies are compared over time. Sampling different times of day may be warranted for sampling multiple life stages (e.g. adults vs. juveniles) of river herring. Knowing the diurnal patterns of juvenile river herring can help to further develop a standardized method for using non-invasive eDNA approaches to assess annual juvenile herring movement.

## Methods

Sample Collection

Daytime and nighttime water samples were collected within the same 24 hr period on the Roanoke River in Plymouth, North Carolina ( Figure 1C, inset) between September 2022 and November 2022 when juvenile river herring are known to be in the river system. A total of 24 water samples were collected (8 daytime, 8 pre-electrofishing nighttime, and 8 post-electrofishing nighttime) ( Figure 1C, green triangle). Samples were collected on September 21st, 2022, October 5th, 2022, October 19th, 2022, and November 10th, 2022. At the time of collection of nighttime samples, electrofishing was conducted to develop catch data to confirm that river herring were present in the system at the time of sampling (Reynolds and Dean, 2020). A total of two eDNA samples were collected at night on the same day varying by the time in which they were collected. Specifically, PRE samples were collected 15-20 minutes before electrofishing and POST samples were collected 15-20 minutes after electrofishing. Water samples were collected in 2-L bottles that had previously been treated with alconox and rinsed with bleach to remove any non-target eDNA or DNAses. On site, bottles were rinsed three times with river water before collecting the water for eDNA analyses and were immediately kept cold with ice packs after water sample collection had occurred (Lee et al., 2024). After transporting back to the lab, 2000 mL of water samples were immediately vacuum-filtered using Nalgene Rapid-Flow Filters (Thermo Fisher Scientific, Waltham, MA, USA) with 0.45 µM cellulose nitrate filters. Filters were then removed and stored in a -80°C freezer to prevent DNA degradation (Yamanaka et al., 2016) until downstream processing.

Electrofishing

Boat-mounted electrofishing was conducted just above the public boating access adjacent to Weyerhaeuser Company in Plymouth N.C. This location was chosen with help from the North Carolina Wildlife Resourses Commission as it was identified as a location where juvenile river herring had previously been sampled as by-catch while sampling for other species at night in the same area. Sampling was completed in accordance with NCWRC Permit 22-SFC00270. Sampling occurred on the same dates in which eDNA samples were taken. An electrofishing unit (Smith-Root GPP) using ~150-200 volts, 4–5 amps, and 60-Hz pulsed DC with two dip netters to collect juvenile Alewife and Blueback Herring. One transect was performed along the bulkhead of the Weyerhaeuser plant for 900 s electrofishing time covering approximately 620 meters. The location sampled during each outing is shown on the map in Figure 1C (orange line). River herrings were netted, identified by species, measured for total length to the nearest millimeter (TL; mm), and weighed to the nearest gram (g). Catch data is shown in Figure 1A .

DNA Extractions and Quantitative PCR (qPCR)

DNA was extracted from the filters using the DNEasy PowerSoil Pro (Qiagen, Hilden, Germany) DNA extraction kit following the manufacturer’s protocol (Feng et al., 2023). This kit was used because it produces high DNA yields for these turbid, organic rich waters. Extracted DNA was then quantified using a quantitative PCR (qPCR) protocol developed by Plough et al. (2018). These primers target part of a mitochondrial gene, cytochrome c oxidase subunit 1 ( *cox1* ) which is specific to both Alewife and Blueback Herring, equally amplifying DNA from both species (Plough et al., 2018). Briefly, DNA samples, standards, and negative controls were run in triplicate 20 µL reactions using the Bio-Rad SYBR Green Master Mix (Bio-Rad, Hercules, CA, USA) and the primers CO1_RH-F (5’- ATG AGC TTC TGA CTA CTT -3’) and CO1_RH-R (5′- GAT AGT TAG ATC GAC GGA -3′) on the Bio-Rad CFX96 Real Time Thermocycler. PCR thermal cycler conditions were 1 cycle of 45 seconds at 95°C, followed by 39 cycles of 5 seconds at 95°C, 15 seconds at 58°C and 10 seconds at 72°C. There were three technical replicates of each sample, standards, and negative controls in every run. Quantitative PCR (qPCR) standards were developed using fin-clips of blueback herring with concentrations spanning several orders of magnitude to ensure accurate quantification and positives were confirmed within this range of all technical replicates. Results were analyzed using Bio-Rad’s CFX Maestro software (version 2.3) to quantify the concentration of river herring DNA in collected water samples and were normalized to ng river herring eDNA per liter of water filtered.

Statistical Analyses & Data Visualization

Statistical analyses were completed to compare day and night eDNA concentrations using non-parametric Kruskal-Wallis Tests and a Generalized Linear Model (GLM, Gaussian Distribution) using base R. Boxplots and scatterplots were generated in R Studio (version 2023.12.1) using the packages ggplot2 (version 3.5.0) and dbplyr (version 2.4.0) for data manipulation.
